# Mental health is strongly associated with capability after lower extremity injury treated with free flap limb salvage or amputation

**DOI:** 10.1007/s00068-024-02459-1

**Published:** 2024-01-30

**Authors:** David D. Krijgh, Teun Teunis, Emile B. List, Marc A. M. Mureau, Antonius J. M. Luijsterburg, Wiesje Maarse, Pascal P. A. Schellekens, Falco Hietbrink, Tim de Jong, J. Henk Coert

**Affiliations:** 1 Department of Plastic and Reconstructive Surgery, University Medical Center Utrecht, Heidelberglaan 100, Postbus, 85500, Postcode 3508 GA Utrecht, the Netherlands; 2 Department of Plastic Surgery, University Pittsburgh Medical Center, Pittsburgh, USA; 3 Department of Plastic and Reconstructive Surgery, University Medical Center Rotterdam, Erasmus MC, Rotterdam, the Netherlands; 4 Department of Trauma Surgery, University Medical Center Utrecht, Utrecht, the Netherlands; 5 Department of Plastic, Reconstructive and Hand Surgery, Radboud University Medical Center, Nijmegen, the Netherlands

**Keywords:** Quality of life, Lower leg, Free flap, Reconstruction, Amputation

## Abstract

**Background:**

Knowledge about factors associated with long-term outcomes, after severe traumatic injury to the lower extremity, can aid with the difficult decision whether to salvage or amputate the leg and improve outcome. We therefore studied factors independently associated with capability at a minimum of 1 year after amputation or free flap limb salvage.

**Methods:**

We included 135 subjects with a free flap lower extremity reconstruction and 41 subjects with amputation, between 1991 and 2021 at two urban-level 1 trauma centers with a mean follow-up of 11 ± 7 years. Long-term physical functioning was assessed using the Physical Component Score (PCS) of the Short-Form 36 (SF36) and the Lower Extremity Functional Scale (LEFS) questionnaires. Independent variables included demographics, injury characteristics, and the Mental Component Score (MCS) of the SF36.

**Results:**

Greater mental health was independently and strongly associated with greater capability, independent of amputation or limb reconstruction. Mental health explained 33% of the variation in PCS and 57% of the variation in LEFS. Injury location at the knee or leg was associated with greater capability, compared to the foot or ankle. Amputation or limb reconstruction was not associated with capability.

**Discussion:**

This study adds to the growing body of knowledge that physical health is best regarded through the lens of the bio-psycho-social model in which mental health is a strong determinant. This study supports making mental health an important aspect of rehabilitation after major lower extremity injury, regardless of amputation or limb salvage.

## Introduction

### Background

The incidence of open tibia fractures is 3.4 per 100,000, most frequently involving young males and older females [1]. Open fractures and other severe lower extremity injuries are often accompanied by loss of soft tissue. Adequate soft tissue coverage is necessary for a functional extremity. Due to a shortage of soft tissue on the lower extremity, free tissue transplantation is often necessary to provide soft tissue coverage of the (injured) bony structures. Alternatively, amputation of the leg can be considered.

### Rationale

After a severe traumatic injury to the lower leg, it is a difficult decision for both the patient and the surgeon whether to amputate or attempt to salvage the leg through free soft tissue transplantation.

Over the last two decades, free tissue transplantation techniques have advanced significantly and have become common practice for many reconstructive microsurgeons. Amputating the leg is a less complex operation, but results in loss of the leg. Compared to amputation, successful limb salvage through free tissue transfer results in a higher level of functional outcomes and self-esteem [2–4]. However, microsurgical lower leg reconstruction is associated with higher levels of complications, re-operations, and a longer hospital stay compared to amputation [5–7]. Conversely, amputation results in a shorter hospital stay and rehabilitation, allowing the patient to return to work sooner and recent advances in prostheses have shown promising results in improved neurological and myoelectric control of the prosthesis [5, 8]. However, patients who have had an amputation are prone to suffer from chronic (phantom) pain as well as physical and mental limitations [5, 9–14].

Although multiple studies in the past have addressed the question whether to reconstruct or amputate, the answer remains unclear [5, 15–18]. Knowledge about which factors are associated with long-term outcomes can aid with this difficult decision and might improve rehabilitation after major lower extremity trauma [19].

### Study questions

We therefore asked: what variables are independently associated with capability measured through the Physical Component Summary (PCS) of the Short Form 36 (SF-36) at a minimum of 1 year after lower extremity injury treated with free flap limb salvage or amputation? Secondarily, we assessed which variables are independently associated with physical function measured through the Lower Extremity Functioning Scale (LEFS) at 1 year.

## Methods

### Study design and setting

All adults who had a posttraumatic free flap lower limb reconstruction or a lower extremity amputation at two urban-level 1 academic medical centers were retrospectively identified. Search periods included 1993 to 2014 at the Erasmus Medical Center Rotterdam and 1991 to 2021 at the University Medical Center Utrecht. Both acute injuries and chronic defects, such as unstable skin, fistulas, and chronic osteomyelitis, were included. At the Erasmus Medical Center, only patients who primarily were reconstructed with a free flap were included in this database. At the UMC Utrecht, patients who either had a reconstruction or an amputation were included. Both centers possessed the skill and ability to perform free flap reconstruction during the complete study period. The decision to proceed with amputation or attempt to salvage the leg through free soft tissue transplantation was a shared decision made by the patient and treating physicians jointly. Patients with a defect due to malignancy, diabetes, vascular insufficiency, pressure ulcers, or an acute infection were excluded. Patients without up-to-date contact details were excluded. Amputation levels above the knee and bilateral amputations were also excluded.

### Participants

**Fig. 1:**
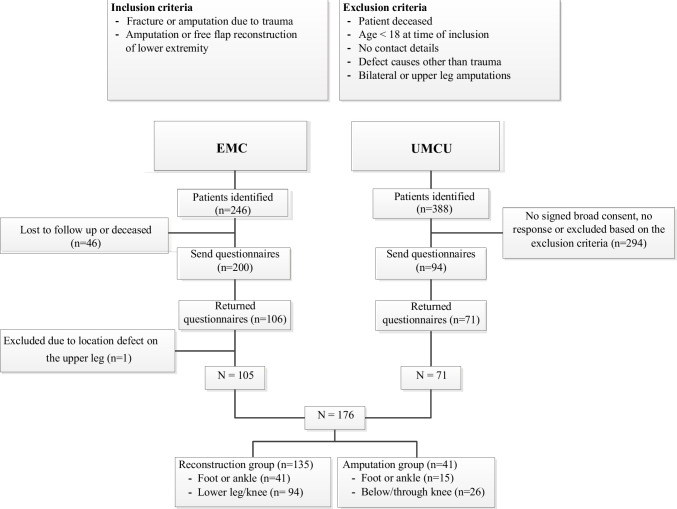
Flowchart of the responders

We identified 598 potential participants (Fig. 1). Of those, 294 (49%) provided written informed consent to participate in the study, and 176 (29%) filled out the provided questionnaires that were sent to them by mail (our final cohort). In case patients did not return the filled-out questionnaires, we contacted them twice by phone to obtain consent.

### Descriptive data

Mean age was 53 years (SD, 15 years), and 39 (22%) were female. Mean follow-up was 11 ± 7 years (Table 1).

**Table 1:** Patient demographics

Demographics	Overall	Amputation	Reconstruction	*P* value
Participants	176	41	135	
Male	137	34 (83%)	103 (76%)	0.52
Age at time of surgery (years)	43±16	42±16	43±16	0.23
Age at time of study (years)	53±15	55±15	52±16	0.76
Hospital				**<0.001**
Erasmus Medical Center	105	11	94	
University Medical Center Utrecht	71	30	41	
Days admitted to hospital	35±25	38±25	35±25	0.42
Tobacco use				
No	102 (58%)	16 (39%)	86 (64%)	**<0.001**
Yes	49 (28%)	10 (25%)	29 (29%)	
Unknown	25 (14%)	15 (37%)	10 (7%)	
Cardiovascular history	19 (11%)	11 (27%)	8 (6%)	**0.001**
Gustilo				0.32
Closed, grade 1 or 2	23 (13%)	6 (15%)	17 (13%)	
3a	23 (13%)	5 (12%)	18 (13%)	
3b	55 (31%)	8 (20%)	47 (35%)	
3c	11 (6%)	4 (10%)	7 (5%)	
Unknown	64 (36%)	18 (44%)	46 (34%)	
Trauma location				0.45
Foot or ankle	56 (32%)	15 (37%)	41 (30%)	
Cruris or knee	120 (68%)	26 (63%)	94 (70%)	
Amputation level				
Through-knee	7 (17%)	7 (17%)	-	
Trans-tibial	24 (59%)	24 (59%)	-	
Distal: ankle or foot	10 (24%)	10 (24%)	-	
SF-36 Mental Component Scale	46±12	47±12	46±12	0.69
LEFS	47±17	42±18	48±17	0.10
SF-36 Physical Component Scale	41±12	40±12	42±12	0.31

Continuous variables as mean (±standard deviation); discrete variables as number (percentage). Bold indicates statistically significant difference

*SF-36* Short-Form 36 (quality of life), *LEFS* Lower Extremity Functional Scale

### Outcome measures

The primary outcome measure of physical function was the PCS of the SF-36 (2^nd^ version) [20, 21]. The 36-Item Short Form is a self-reported questionnaire for health-related quality of life, containing eight different scales and two component scores: the Physical Component Score (PCS) and the Mental Component Score (MCS). This score ranges from 0 to 100, with a higher score indicating greater physical function. The Physical Component Summary (PCS) and the Mental Component Summary (MCS) were calculated in an oblique fashion using the normative SF-36 scores for the Dutch Population and the coefficients for the Dutch population [20–24].

As a secondary outcome, lower extremity specific physical function was assessed using the LEFS [25]. The LEFS is a questionnaire with 20 items on the functional impairment of one or both lower extremities, which has the ability to discriminate between pain and functioning of the lower limb. Scale scores vary between 0 and 80, with higher scores meaning better limb functioning. Both questionnaires were validated in Dutch [20, 26].

### Other measures

Comorbidities, medication use, and tobacco use were retrieved from the electronic medical files. If available, the Gustilo Anderson classification was recorded [27].

### Ethical approval

This study was approved by the CCMO Dutch Medical Research Ethics Commission (reference number Utrecht 16-291 and Rotterdam 2015-542).

### Statistical analysis

Potential differences in baseline characteristics (Table 1) and possible variables associated with our outcome variables (PCS and LEFS) were identified using bivariate analysis (Table 2). Variables with *P*<0.10 on bivariate analysis were entered into multivariable analysis in addition to type of reconstruction (primary variable of interest). We assessed potential collinearity through the variable inflation factor. All analyses were performed using StataCorp LLC., College Station, TX. Since this is a retrospective study, we did not perform a power analysis. There was no missing data. We tested the association between time and our independent variables (LEFS and PCS) to assess if advances in care and technology resulted in a confounding effect. We found no association (*r* LEFS 0.006, *P* 0.94 and PCS –0.59, *P* 0.45).

**Table 2:** Bivariate analysis of factors associated with physical function

Variable	LEFS	*P* value	PCS	*P* value
Sex				
Male	46±17	0.45	41±11	0.83
Female	48±19		42±13	
Age at time of surgery (years)	-0,093	0.22	-0.096	0.23
Age at time of study (years)	-0,14	0.067	-0,12	0.11
Hospital				
Erasmus Medical Center	47±18	0.99	41±12	0.86
University Medical Center Utrecht	47±16		42±11	
Days admitted to hospital	-0.031	0.70	-0,035	0.66
Tobacco use				
No	49±17	0.087	43±11	0.24
Yes	45±16		40±12	
Unknown	41±18		39±13	
Cardiovascular history				
No	47±17	0.97	41±12	0.55
Yes	46±17		43±13	
Gustilo				
Closed, grade 1 or 2	49±14	0.78	44±10	0.91
3a	49±21		42±13	
3b	46±17		41±11	
3c	50±15		40±12	
Unknown	45±17		41±12	
Trauma location				
Foot or ankle	43±17	0.070	39±12	0.15
Cruris or knee	48±17		42±12	
SF-36 Mental Component Scale	0.57	**<0.001**	0.75	**<0.001**
Surgery				
Amputation	43±18	0.10	40±12	0.31
Reconstruction	48±17		42±12	

Bold indicates statistically significant difference; continuous variables as mean (±standard deviation); discrete variables as number (percentage)

## Results

### Variables independently associated with Physical Component Score

The only variable independently associated with greater physical function was greater mental health (MCS) (beta 0.72, 95% CI 0.62 to 0.82, *p*<0.001). Mental health accounted for 57% of the variation in lower extremity physical function. Interestingly, type of reconstruction was not associated with physical function (Table 3).

**Table 3:** Multivariable analyses of factors independently associated with physical function

Variable	Regression coefficient	Standard error	*P* value	VIF	Semi-partial *R*^2^	Adjusted *R*^2^
	(95% confidence interval)					
*Physical Function Scale*						0.57
SF-36 Mental Component Scale	0.72 (0.62 to 0.82)	0.050	**<0.001**	1.02	0.57	
Surgery type						
Amputation	Reference value					
*Lower Extremity Functional Scale*						
Trauma location						0.39
Foot or ankle	Reference value					
Cruris or knee	5.7 (0.9 to 10)	2.4	**0.02**	1.07	0.02	
SF-36 Mental Component Scale	0.81 (0.63 to 0.99)	8.9	**<0.001**	1.03	0.33	
Surgery type						
Amputation	Reference value					
Reconstruction	3.9 (–1.9 to 9.7)	2.9	0.18	1.35		

Multivariable analysis of factors that are independently associated with physical functioning corrected for hospital, tobacco use, cardiovascular disease, Gustilo classification, trauma location, and age. Only the semi-partial *R*^2^ of significant values is displayed. Bold indicates statistical significance, *P* < 0.05

*LEFS* Lower Extremity Functional Scale questionnaires, *PCS* Physical Component Score, *CI* confidence interval, *VIF* variance inflation factor, *SF-36* Short-Form 36

### Variables independently associated with Lower Extremity Physical Function Scale

Trauma location was independently associated with LEFS, with proximal lower leg injuries having more favorable physical function compared to foot or ankle trauma (beta 5.7, 95% CI 9.9 to 10, *P*=0.02); however, location only accounted for 2% of the variation in physical function. Better mental health was strongly associated with better physical function (beta 0.81, 95% CI 0.63 to 0.99, *P*<0.001) and accounted for 33% of the variation. Patients did not differ in physical function after amputation or reconstruction (Table 3).

## Discussion

The decision to attempt to salvage or instead amputate a severely injured lower leg can be a difficult one to make for both surgeon and patient [15, 18]. Knowledge of potentially modifiable variables influencing outcomes after limb salvage or amputation can improve treatment decision making and can help optimize physical function. We found no difference in physical function between limb salvage or amputation. Instead, we found a strong association between physical function and mental health. This finding emphasizes the bio-psycho-social model of health and offers a potentially modifiable variable (mental health) to improve physical function after major lower extremity trauma.

The following limitation should be noted: First, this is a cross-sectional study. A randomized controlled study of limb salvage and amputation is impractical and unethical. Unobserved differences between people having free tissue transplantation and amputation might drive the decision for treatment, and their later physical function. We accounted for a large number of important variables through multivariable analysis to minimize the confounding effect of any unobserved variables. Second, we were only able to contact a relatively small proportion of the total number of people treated at our institutions (176/598, 29%). However, our study questions tested the strength of association (prognostic variables), which depends on variation in the data, not on response rate. Third, we could only test association. It is unclear whether less physical function caused reduced mental health or the other way around. A previous study found that greater symptoms of anxiety and depression at the preceding time point were associated with reduced capability, suggesting a causal response where reduced mental health leads to reduced capability [28–30]. Fourth, due to the retrospective nature of our study, we were not able to include all possible variables associated with capability. A future study might include adjustment to an artificial limb (although this might be a mediator between mental health and capability [31]), cognitive impairment, chronic vs. acute injuries, or household income. Regardless, we expect that even if these variables were included, this would not change the relatively strong association between mental health and capability found in this, and other studies [32].

We found a strong association between greater capability, measured by two different questionnaires, and better mental health. Our findings are in line with a prior study of 327 patients with a lower extremity injury that found that greater symptoms of depression and anxiety increased the negative effect of pain on capability up to 1 year after injury [30]. At 2 years after injury, pain intensity was no longer associated with capability, but greater symptoms of depression and anxiety still limited capability. This suggests that over time the relationship between mental health and capability increases. Our study results suggest this relationship remains preserved at a mean of 11 years after injury. There are various prior studies emphasizing a strong association between mental health and capability after fracture [28–30]. A study of 385 participants with lower limb injuries showed that 42% (*n*=161) suffered from a potential psychological disorder measured through screening questionnaires 2 years after injury and almost one-fifth of the people reported to have severe phobic anxiety and/or depression [28]. Wegener et al. found that higher levels of negative affect predict lower levels of functioning at subsequent periods during recovery from lower extremity trauma, and they concluded that more attention needs to be paid to issues like anxiety and depression to maximize the function and quality of life in persons with injuries [30]. Our results suggest that unhelpful thoughts and feelings offer a potentially modifiable variable to improve capability after severe lower extremity injury, potentially years after the injury. A meta-analysis assessing risk factors for developing affective disorders after open lower limb fracture concluded that in addition to the medical and surgical care, the psychological needs will need to be addressed in order to deliver holistic care [33]. Positive effects on recovery, following appropriate management of the psychological illness, have already been observed for head injuries and critical illness [34, 35]. Our current study further supports in implementing psychological support as standard of care to patients with a severe lower leg injury. A future study can assess if addressing mental health leads to improve physical function.

We found a somewhat greater capability (measured by PCS) in people with a more proximal injury (cruris or knee) compared to ankle or foot injuries. We could not replicate this finding when using LEFS as our dependent variable. This finding suggests that people with a more proximal lower leg reconstruction or amputation physically perform somewhat better than people with a reconstruction or amputation at the level of the ankle or the foot. Out of the 41 amputations performed in our cohort, 10 were at the level of the foot/ankle, 24 transtibial, and seven through-knee. There is some evidence that a transtibial amputation is related to a higher quality of life than a through-knee amputation [3]. Braaksma et al. concluded in their systematic review there are not enough comparative studies of adequate quality to directly compare ankle/foot amputations and transtibial amputations [36]. An amputation at the ankle or foot level leaves a longer lower leg stump which complicates prosthetic fitting [37]. This finding is supported by a prospective cohort study of 569 people with lower leg injury at level 1 trauma centers that indicated that a foot amputation was associated with poor outcome [17]. In case of a reconstruction, our finding of a greater capability in patients with more proximal injuries may be related to the difficulties associated with a reconstruction of the weight-bearing area of the foot. Decreased sensation in the sole of the foot frequently results in challenges wearing normal shoes and due to the fact that patients often have a changed anatomy of the foot and thicker areas due to the free flap reconstruction, custom-made shoes are frequently indicated.

We found no difference in quality of life between patients with a free flap lower extremity reconstruction and amputation. Within the literature, there is still discussion about whether an amputation or salvaging the lower extremity results in better outcomes. Due to the heterogeneity of the injuries in this patient group, it is often very difficult to compare patients who have had an amputation to patients who have had a reconstruction. Many studies, including the largest study to date—the LEAP study—show that the outcomes of patients who have had an amputation vs. a reconstruction are comparable [5, 16, 17]. Despite the fact that reconstruction is not clearly favorable over amputation, people generally indicate a preference for reconstruction [18, 38]. A study in patients with an amputation indicated that mobility is a strong independent predictor for a higher quality of life and satisfaction [39]. Besides looking at physical capability, a recent study by Korozumi et al. compared the mental health status of patients with an amputation to patients who have had a reconstruction. They concluded that limb salvage resulted in greater capability and mental health than amputation [40]. Although our current study is not able to reproduce the findings of Korozumi et al., we believe in the importance of including mental health early on into the rehabilitation of patients with severe lower extremity injuries. A collaboration between the trauma department, the rehabilitation department, and the psychology and/or psychiatry department would be the cornerstone of providing mental health support to this patient group in a standardized way.

## Conclusion

This study adds to the growing body of knowledge that physical health is best regarded through the lens of the bio-psycho-social model in which mental health is a strong determinant. This study supports making mental health an important aspect of rehabilitation after major lower extremity injury, regardless of amputation or limb salvage. The relationship seems present even many years after injury. Future studies can assess if improving mental health results in improved physical functioning and quality of life after major lower extremity injury.
